# The effects of iodine blocking following nuclear accidents on thyroid cancer, hypothyroidism, and benign thyroid nodules: design of a systematic review

**DOI:** 10.1186/s13643-015-0106-3

**Published:** 2015-09-24

**Authors:** Steffen Dreger, Manuela Pfinder, Lara Christianson, Stefan K Lhachimi, Hajo Zeeb

**Affiliations:** Department of Prevention and Evaluation, Leibniz Institute for Prevention Research and Epidemiology BIPS GmbH (BIPS), Achterstrasse 30, PO Box 28359, Bremen, Germany; Collaborative Research Group for Evidence-Based Public Health, Leibniz Institute for Prevention Research and Epidemiology BIPS GmbH (BIPS), Bremen, Germany; Institute for Public Health and Nursing Research, Health Sciences Bremen, Bremen University, Bremen, Germany; Health Sciences Bremen, Bremen University, Bremen, Germany

**Keywords:** Stable oral iodine, Potassium iodine, Chernobyl, Health outcomes, I-131, Reactor accident

## Abstract

**Background:**

One of the most efficient radiation protection methods to reduce the risk of adverse health outcomes in case of accidental radioactive iodine release is the administration of potassium iodine (KI). Although KI administration is recommended by WHO’s guidelines for iodine prophylaxis following nuclear accidents and is also widely implemented in most national guidelines, the scientific evidence for the guidelines lacks as the guidelines are mostly based on expert opinions and recommendations. Therefore, this study will provide evidence by systematically reviewing the effects of KI administration in case of accidental radioactive iodine release on thyroid cancer, hypothyroidism, and benign nodules.

**Methods:**

We will apply standard systematic review methodology for the identification of eligible studies, data extraction, assessment of risk of biases, heterogeneity, and data synthesis. The electronic database search will be conducted in MEDLINE (via PubMed) and EMBASE, and covers three search blocks with terms related to the health condition, intervention, and occurrence/location. We have no date or language restrictions, but restrictions to humans only. We will include studies comparing the effects of KI administration on thyroid cancer, hypothyroidism, and benign thyroid nodules in a population exposed to radioactive iodine release. The quality of the studies will be graded. If feasible, a meta-analysis will be conducted.

**Discussion:**

This proposed systematic review will update the existing WHO guideline from 1999. New evidence on the efficacy of KI administration to reduce thyroid cancer, hypothyroidism, and benign thyroid nodules in the event of an accidental release of radioactive iodine to the environment will provide the basis for an update of the WHO guideline for iodine prophylaxis following nuclear accidents.

**Systematic review registration:**

PROSPERO CRD42015024340

**Electronic supplementary material:**

The online version of this article (doi:10.1186/s13643-015-0106-3) contains supplementary material, which is available to authorized users.

## Background

### Description of the condition

Radioactive isotopes of iodine (I-131) are generated in large amounts as a by-product of uranium fission, which is primarily used in nuclear reactors for energy production. In the event of a nuclear reactor accident and when radioactive material is released to the atmosphere, I-131 may be incorporated into the human body through inhalation or ingestion of contaminated food and milk [1]. When inhaled, about 10–30 % of the radioactive iodine will primarily accumulate in the thyroid gland while the remaining amount will be discharged from the body with the urine [2]. As part of I-131’s decay process, beta-radiation is emitted and affects the thyroid and its surrounding tissue and may lead to adverse health outcomes such as thyroid dysfunctions and thyroid cancer.

From the Life Span Study, there is evidence for the development of benign and malignant thyroid nodules as a result of external exposure to ionizing radiation among the atomic bomb survivors (e.g., [3]). Following the Chernobyl reactor accident, which involved a large release of I-131 into the environment, significantly increased numbers of thyroid cancer and thyroid dysfunction such as hypothyroidism were observed in individuals from highly contaminated regions in Ukraine and Belarus [4–6]. In addition, children and adolescents have been found at higher risk for developing thyroid diseases compared to adults. This is due to their smaller thyroid gland, its development during childhood and adolescence which leads to a five to tenfold increase of committed thyroid dose, higher uptake of radioiodine, and higher sensitivity to radioiodine release of the organs, tissues, and cells [7–9]. Further, it is suggested that radiation exposure during the prenatal phase is associated with an increased risk for thyroid cancer [10], and I-131 transmission from mother to infant during breastfeeding has been investigated as an additional risk factor for infants to develop thyroid cancer in later stages in life [11, 12]. In contrast, radiation-induced thyroid cancer risk for adults is thought to be very low and may be close to zero [13].

### Description of the intervention and how it might work

The oral administration of potassium iodine (KI) is assumed to be the most effective and preventive radiation protection measure to reduce the risk of adverse health outcomes for the exposed population in the event of an accidental release of radioactive iodine [14, 15]. KI essentially saturates the iodide transport mechanism of the thyroid by inhibiting the intrathyroid organification of iodide (acute Wolff-Chaikoff effect), by dilution and by promoting excretion and thus, reducing the amount of committed dose to the thyroid gland, its surrounding tissue, and the body [16–18].

KI administration depends on the predicted exposure levels to the thyroid of the defined population groups (i.e., intervention/action levels). KI doses further vary to account for the respective risks of vulnerable population groups (newborn, children and adolescents, and pregnant and lactating women). The effective blocking of the thyroid is achieved with a dose of 130–170 mg of potassium iodine. Fractions of these quantities are to be used in specific population groups (1 in adults, adolescents in addition to pregnant and lactating women, if necessary; 1/2 in children; 1/4 in infants; 1/8 in newborn) [19, 20]. Although KI administration blocks the thyroid gland, it does not provide complete protection from accumulating radioactive iodine. A single dose of KI approximately blocks the thyroid between 24 and 36 h but the blocking capacity decreases with increased time after administration [19, 21]. In the event of continuous release of I-131, repeated administration may be required to ensure prolonged protection of the general population as the protective effect of one KI dose decreases with time.

The Polish government initiated KI administration in the Polish general population, in particular in children and adolescents, in late April and early May 1986 as a consequence of the reactor accident in Chernobyl and the subsequent discharge of radioactive iodine to the environment. Assessing the efficacy of the KI administration, Nauman and Wolff [22] estimated a reduction in committed thyroid dose between 40 and 62 % for those children who were administered KI 1–4 days after the start of exposure. With regard to the timing of the intervention, a simulation study demonstrated higher protective KI efficacy when its administration is carried out in early exposure stages (78.9 vs. 39.1 % with KI given within 2 h or at 8 h after uptake of radioactive iodine, respectively) [15]. It is notable that in Poland as a result of the immediate thyroid-blocking measures implemented within the first 4 days after the start of the exposure, it was achieved that about 90 % of the children under the age of 16 showed thyroid dose commitments below the predicted mean maximal burden (<50 mSv) in this risk group [22].

A recent systematic review further examined the adverse side effects of KI administration to block the thyroid [23]. The evidence gathered from the systematic review suggested that even the administration of high doses of KI did not result in serious adverse health outcomes in the exposed population groups. Severe reactions of clinical significance were rare and in particular observed in individuals with pre-existing thyroid disorders and iodine sensitivity. There was little data available on age differences. The review results however suggested that newborns and the elderly may experience more adverse side effects after KI administration compared to other age groups [23]. Overall, the evidence base was relatively weak because with the exception of the Polish study by Nauman and Wolff [22], most studies on the effects of KI were primarily set in the clinical context and addressed exposure reduction as part of therapy procedures.

### Why it is important to do this review

Iodine thyroid blocking using potassium iodine (KI) is regarded as the most effective radiation protection measure in the event of an accidental release of radioactive iodine to reduce the risk of adverse health outcomes for exposed populations. KI administration is endorsed by WHO’s guidelines for iodine prophylaxis following nuclear accidents and is also widely implemented in most national guidelines. To date, the current guidelines are primarily based on expert knowledge and opinion while the scientific base was not established and reviewed systematically.

As part of the update of the existing WHO guideline from 1999 [20], present WHO regulations for guidelines development require a systematic review of the scientific evidence in order to inform the updating process [24]. Thus, the present project aims to provide an up-to-date review on the efficacy of KI administration to reduce adverse health outcomes such as thyroid dysfunctions and thyroid cancer for the general population in the event of an accidental release of radioactive iodine to the environment.

### Objectives

We aim to assess the effects of KI administration on thyroid cancer, hypothyroidism, and benign thyroid nodules in a population exposed to radioiodine release.

In particular, it is necessary to assess whether specific population groups (e.g., children and adolescents between 0 and 18 years of age, pregnant or lactating women) are differentially affected by KI administration, and to identify appropriate timing, and in circumstances of repeated/continuous exposure, whether repeated KI administration may be warranted to reduce the accumulation of I-131 in the thyroid gland in the exposed population.

## Methods

### Criteria for considering studies for this review

#### Types of studies

The review intends to cover a broad spectrum of research questions that are not necessarily assessed in randomized clinical trials (RCTs). Thus, non-randomized studies will be included in the review. More specifically, the following experimental and observational study types will be covered:

- RCTs
- Quasi-RCTs
- Controlled before-after studies
- Time-series
- Cohort studies
- Case-control studies
- Surveys, e.g., pharmacoepidemiological studies

#### Types of participants

Participants included in studies are either the general population or workers. No further specification is feasible. The literature search is limited to evidence from studies in humans.

#### Types of interventions

The interventions to be evaluated arise from the objectives as outlined above.

The following intervention is considered:

- Stable oral iodine/potassium iodine administration in the general population exposed to external ionizing radiation or radioactive iodine in the environment.

#### Types of outcome measures

The review will include studies that report the following outcome measures:

- Prevalence and incidence of radiation-induced thyroid cancer,
- Prevalence and incidence of radiation-induced hypothyroidism,
- Prevalence and incidence of radiation-induced benign thyroid nodules, and
- Mortality from radiation-induced thyroid cancer (hypothyroidism and benign thyroid nodules are not considered to be associated with mortality).

### Search methods for identification of studies

#### Electronic searches

We will search the following academic databases:

- MEDLINE (1946 to present)
- Excerpta Medica Database (EMBASE) (1947 to present)

We will apply a search strategy with additional keywords for possible comparators and we will not use filters for study types to improve the results of the literature search with respect to the total number of relevant studies.

The databases as listed above will be searched until 16 June 2015.

For details on the MEDLINE and EMBASE search strategies, see Additional files 1 and 2, respectively.

### Searching other resources

All relevant records for additional relevant studies will be searched by hand.

### Advisory group

We have established a review advisory group of experts in the field of thyroid cancer, iodine thyroid blocking and systematic reviews to further comment and provide advice and suggestions to improve the manuscript in protocol and review stages.

In protocol stage, Christoph Reiners, Rita Schneider (UK Würzburg), Elie Akl (AUB, Beirut), Zhanat Carr, and Susan Norris (both WHO) have provided feedback on the research questions. Tomas Allen (WHO) has provided feedback on the search strategy and the selected databases. Vladimir Saenko (Nagasaki University) supported the literature search.

### Data collection and analysis

#### Selection of studies

A research librarian will assist the database search for relevant studies. First, studies’ titles and abstracts, if feasible, as identified by the search will be reviewed by two authors independently. Second, both reviewers will compare their list of relevant studies, and in case of any disagreement, the opinion of a third author will be decisive. Additionally, a third author screens the list of relevant studies. Third, full texts of potentially relevant studies will be retrieved or obtained. Fourth, the full texts will be screened by the reviewers independently. Fifth, each reviewer will create a list with studies that are considered to fulfill the inclusion criteria. Sixth, the reviewers will compare their list with each other, and in case of any disagreement, the opinion of a third author will be decisive.

Based on these six steps, studies will be included for the review. A flowchart based on the preferred reporting items for systematic reviews and meta-analyses (PRISMA) will be developed to visualize the selection of included studies. Moreover, we will provide a table with statements on excluded studies.

### Data extraction and management

Data extraction will be performed by two authors independently. In case of any disagreement, the opinion of a third author will be decisive. We will use a modified data extraction and assessment template from the Cochrane Public Health Group (CPHG). Previous to the major data extraction process, the authors will pilot the data extraction form to ensure a standardized extraction. We will extract general information (publication type, country of study, funding source of study, and potential conflict of interest from funding), study eligibility (type of study, participants, type of intervention, duration of intervention, and type of outcome measures), and study details (study intention, methods, results, intervention group, outcomes, and other relevant information).

Data will be assembled and inserted into RevMan 5.3 by one author.

### Assessment of risk of bias in included studies

The risk of bias of every included study will be evaluated by two authors independently. In case of any disagreement, the opinion of a third author will be decisive. Based on the template provided from the CPHG, the risk of bias will be assessed using the criteria for judging risk of bias in Cochrane’s “Risk of bias” assessment tool and the Cochrane Group’s Effective Practice and Organisation of Care (EPOC) guidance for interrupted time series (ITS) tool. Cochrane’s “Risk of bias” assessment tool and the EPOC risk of bias tool for ITS examine the following biases: selection, performance, detection, attrition, reporting, and others. The EPOC risk of bias tool for ITS examines three further risks of bias: “Was the intervention independent of other changes?,” “Was the shape of the intervention effect pre-specified?,” and “Was the intervention unlikely to affect data collection?”

In the assessment of the risk of bias in cohort studies, we will follow the best practice recommendation to assess the specific features of cohort studies and the extent to which these may introduce bias [25, 26]. At minimum, we will assess the risk of bias in the following features: sampling strategy, response rates, sample representativeness, attrition, participant allocation, exposure assessment, outcome assessment, and reporting and control of key confounders and control of reverse causation.

To judge the risk of bias, the following categories will be used: “no” (risk of bias is low), “yes” (risk of bias is high), and “unclear” (information lacks or uncertainty about the risk of bias) [27].

### Measurement of treatment effect

Data synthesis aims to report changes in outcome measures from baseline to the post-intervention phase. Dichotomous data will be expressed as odds ratios (ORs), risk ratios (RRs), or risk differences (RDs). In accordance with the recommendations from the Cochrane Public Health Group, RRs will be the preferred reported data type. If RRs are not presented in the study, but data to calculate the RRs are provided, we will calculate them. If data to calculate the RRs are not provided, we will contact the corresponding author of the study for the RRs or the data to calculate the RRs by email or phone. If we cannot provide RRs, we will use the data provided in the study to report the treatment effect.

Continuous data will be expressed as standardized mean differences (MDs). Shorter ordinal data will be translated into dichotomous data (expressed as ORs, RRs, or RDs) and longer ordinal data will be treated as continuous data (expressed as the standardized MDs). Count data and Poisson data will be expressed as rate ratios. Time-to-event data (survival data) will be translated into dichotomous data when appropriate or into hazard ratios (HRs).

### Unit of analysis issues

The analysis will consider the level at which randomization occurred, e.g., cluster-randomized trials, cross-over trials, and multiple observations (repeated observations on subjects, recurring events, multiple body parts, and multiple intervention groups) for the same outcome [27].

### Dealing with missing data

We will contact study authors if relevant data is missing. Data “not missing at random” due to publication bias, systematic loss to follow-up, or systematic exclusion of individuals from studies will be identified and requested from study authors.

### Assessment of heterogeneity

In the event of substantial clinical, methodological, or statistical heterogeneity, we will not perform meta-analytic pooling.

Heterogeneity will be detected through visual inspection of the forest plots and by using a standard chi-squared test with a significance level of *P* < 0.1 [27]. The *I*^2^ statistic will be applied to quantify inconsistency across studies and to assess the impact of heterogeneity on the meta-analysis [27]. Potential reasons for heterogeneity will be examined by conducting subgroup analyses.

### Assessment of reporting bias

Reporting biases, including publication bias, time lag bias, multiple (duplicate) publication bias, location bias, citation bias, language bias, and outcome reporting bias, occur when the dissemination of research results depends on their magnitude and direction [27]. Study quality and risk of bias of randomized controlled trials are assessed with the Cochrane risk of bias tool [27]. Study quality and risk of bias of non-randomized quantitative studies are assessed with quality assessment tool for quantitative studies [28]. If feasible, we will apply funnel plots for visual assessment for study effects resulting from reporting biases. When testing asymmetry in funnel plots (small study effects), we will investigate whether the size of the relation between a measure of study size and the estimated intervention effect is larger than it is supposed to be [27]. RevMan 5.3 will be used for the graphical representation of the funnel plots.

### Data synthesis

If feasible, we will perform meta-analyses by applying RevMan 5.3 for study results with clinical, methodological, and statistical homogeneity. For dichotomous outcomes, we will apply the Mantel-Haenszel method, and for continuous outcomes, we will apply the inverse variance method. For all analyses, the random effects method will be applied.

Study results with insufficient homogeneity will be presented in a narrative synthesis.

We will provide a “Summary of findings” table [27]. This table includes information on the outcomes, illustrative comparative risks, the relative effect, the number of participants, the number of studies included, the quality of evidence (GRADE), and additional comments. GRADEprofiler will be used to prepare the “Summary of findings” table.

### Subgroup analysis and investigation of heterogeneity

We will investigate the following subgroups for primary outcomes, where feasible:

- Children and adolescents (0–18 years) versus adults
- Males versus females
- Pregnant and lactating women versus other women
- Dosage of intervention (e.g., low or high)
- Timing of intervention (e.g., before, shortly after, or long after exposure)
- Timing of exposure (e.g., one time, two or more times, continuously)
- Magnitude of exposure (e.g., strong or weak)
- Repetition of intervention (e.g., after single, several, or continuous exposures)

If feasible, we will apply *t* tests and chi-squared tests to investigate statistical significance of between subgroup differences in the treatment effect and we will consider multiple test bias.

### Sensitivity analysis

Sensitivity analyses will be performed to determine the robustness of our results. To assess the impact of risk of bias, we will conduct meta-analyses (if feasible):

- With studies considered as “low risk of bias” and then compare results to those of studies considered as “high risk of bias”
- With “large studies” and then compare the results to those of “small studies”
- With published studies and then compare results to those of unpublished studies

## Discussion

We will perform a somewhat unusual review in a very specific research area to support the further development of a WHO guideline on KI application in nuclear accidents with population exposure to radioactive iodine. Although, there is much research on health consequences of radiation exposure available, not much specific evidence on intervention effects is anticipated, because of mostly unsystematic application of KI in exposed populations so far, and resulting difficulties in researching potential effects at population level. To get a clearer picture on the issue and support the work of the guideline development group, we will aim to derive evidence on the effectiveness of KI intake in subgroups, e.g., pregnant women. In addition, the dosage and the timing of the intervention may be relevant for the effectiveness of KI on thyroid blockade. To our knowledge, this will be the first systematic review that synthesizes information on the effectiveness of KI administration to reduce the risk of thyroid cancer, hypothyroidism, and benign nodules in case of a nuclear accident.

### Strengths and limitations

Our findings will depend on the quality and the number of studies found. We focus on two large literature databases and might thus not include studies that are not included in these databases. However, from our research prior to conducting the review and from consultation with various experts on other potentially relevant databases, this choice seems justified. Some studies might be in Russian, Polish, or Japanese language. However, we have experts from these countries assisting us in translating the content, extracting the data, and discussing quality and other issues.

### Review status

The authors have started searching relevant studies and electronic databases. We expect the review to be completed by October 2015.

## Additional files

Additional file 1:
**MEDLINE/PubMed search.** (DOCX 16.4 kb) Additional file 2:
**EMBASE search.** (DOCX 16.7 kb)

## Abbreviations

CPHG: Cochrane Public Health Group
EMBASE: Excerpta Medica Database
EPOC: Effective Practice and Organisation of Care
HR: hazard ratio
ITS: interrupted time series
MD: mean difference
OR: odds ratio
RD: risk difference
RR: risk ratio
RCT: randomized clinical trial
WHO: World Health Organization
