# Supplementary material for: The effects of iodine blocking following nuclear accidents on thyroid cancer, hypothyroidism, and benign thyroid nodules: design of a systematic review
Source: Syst Rev. 2015 Sep 24;4:126. doi: 10.1186/s13643-015-0106-3 (PMC4588908; doi:10.1186/s13643-015-0106-3)
Supplement: Additional file 1: — MEDLINE/PubMed search. (DOCX 16.4 kb) [file 13643_2015_106_MOESM1_ESM.docx]

# Additional file 1

# Medline/PubMed Search

## Block 1: health conditions

| **Search name** | **Search query** | **Type of search** | **Results** |
| --- | --- | --- | --- |
| 1A(1) | "thyroid gland"[MeSH Terms] OR "hypothyroidism"[MeSH Terms] OR "thyroid diseases"[MeSH Terms] OR "thyroid neoplasms"[MeSH Terms] OR "neoplasms, radiation-induced"[MeSH Terms] OR "radiation dosage"[MeSH Terms] OR "radiation injuries"[MeSH Terms] OR "dose-response relationship, radiation"[MeSH Terms] | MeSH  major & sub-terms | 268.837 |
| 1B | ((thyroid*[Title/Abstract]) AND dysfuntion*[Title/Abstract] OR abnormalit*[Title/Abstract] OR cancer[Title/Abstract] OR cancers[Title/Abstract] OR tumor[Title/Abstract] OR tumour[Title/Abstract] OR tumors[Title/Abstract] OR tumours[Title/Abstract] OR nodul*[Title/Abstract] OR carcinogen*[Title/Abstract] OR carcinoma*[Title/Abstract] OR malignanc*[Title/Abstract] OR medullar*[Title/Abstract] OR metastases[Title/Abstract] OR metastasi*[Title/Abstract] OR enlarged[Title/Abstract] OR disease*[Title/Abstract] OR hypothyroidism[Title/Abstract])) | keyword  TI/AB | 85.439 |
| 1 | 1A(1) OR 1B |  | 292.436 |

## Block 2: intervention(s)

| **Search name** | **Search query** | **Type of search** | **Results** |
| --- | --- | --- | --- |
| 2A(1) | "Potassium Iodide"[Mesh] OR "Iodine Radioisotopes"[Mesh] | MeSH  major terms | 50.555 |
| 2B | ("ITB" OR "iodine thyroid blocking" OR "potassium iodide" OR "Iodine Radioisotope*" OR "KI" OR "sodium iodide" OR ((blockade* OR blocking OR administration) AND iodine) OR "stable iodine" OR ((prophylaxis OR prophylactic* OR "prophylactic agent*") AND (iodine* OR iodide*))) | keyword | 83.987 |
| 2 | 2A(1) OR 2B |  | 124.533 |

## Block 3: occurrence/location

| **Search name** | **Search query** | **Type of search** | **Results** |
| --- | --- | --- | --- |
| 3A | "Radioactive Hazard Release"[Mesh] OR "Radioactive Fallout"[Mesh] OR "Nuclear Warfare"[Mesh] OR "Nuclear Reactors"[Mesh] OR "Chernobyl Nuclear Accident"[Mesh] OR "Nuclear Power Plants"[Mesh] OR "Fukushima Nuclear Accident"[Mesh] | MESH  major terms | 15.430 |
| 3B(3) | ((Nuclear* OR atomic OR reactor* OR radioactive* OR radiation OR radiological*) AND (accident* OR warfare OR contaminat* OR exposure* OR fallout OR meltdown OR disaster* OR catastrophe*)) OR ((Belarus OR chernobyl OR Chornobyl OR Hiroshima OR Fukushima OR Gomel OR Homel OR Ukraine OR Minsk OR "3 mile" OR "three mile" OR Nagasaki OR Pripyat OR Poland OR Russia OR USSR OR "Soviet Union" OR Japan) AND (accident* OR warfare OR contaminat* OR exposure* OR fallout OR meltdown OR disaster* OR catastrophe*)) | keyword | 175.763 |
| 3 | 3A OR 3B(3) |  | 177.762 |

### Limits: publication types, human studies

| **Search name** | **Search query** | **Results** |
| --- | --- | --- |
| 4A | "case reports"[Publication Type] | 1.724.784 |
| 4B | ("case reports"[Publication Type] OR "news"[Publication Type] OR "newspaper article"[Publication Type]) | 1.908.867 |
| 4C | "animals"[Mesh] | 17.833.169 |
| 4D | "humans"[Mesh] | 13.824.418 |

### Summary & results

| **Search name** (Saved in PubMed & EndNote) | **Results** |
| --- | --- |
| 1 AND 2 AND 3 | 1.321 |
| 1 AND 2 AND 3 (AND) NOT 4A | 1.240 |
| 1 AND 2 AND 3 (AND) NOT 4B | 1.225 |
| 1 AND 2 AND 3 (AND) NOT 4A (AND) NOT 4C | 47 |
| 1 AND 2 AND 3 (AND) NOT 4B (AND) NOT 4C | 47 |
| 1 AND 2 AND 3 (AND) NOT 4A AND 4D | 1.038 |
| 1 AND 2 AND 3 (AND) NOT 4B AND 4D | 1.023 |
