# Supplementary material for: The effects of iodine blocking following nuclear accidents on thyroid cancer, hypothyroidism, and benign thyroid nodules: design of a systematic review
Source: Syst Rev. 2015 Sep 24;4:126. doi: 10.1186/s13643-015-0106-3 (PMC4588908; doi:10.1186/s13643-015-0106-3)
Supplement: Additional file 2: — EMBASE search. (DOCX 16.7 kb) [file 13643_2015_106_MOESM2_ESM.docx]

# Additional file 2

# EMBASE search

## Block 1: health conditions

| **Search name** | **Search query** | **Type of search** | **Results** |
| --- | --- | --- | --- |
| 1A | ("thyroid gland" or hypothyroidism or "thyroid disease" or "thyroid tumor" or "radiation induced neoplasm" or "radiation dose" or "radiation injury" or "radiation response").sh. | EMTREE headings  & subheadings | 200.489 |
| 1B | (thyroid* and (dysfuntion* or abnormalit* or cancer* or tumo?r* or nodul* or carcinogen* or carcinoma* or malignanc* or medullar* or metastases or metastasi* or enlarged or disease* or hypothyroidism)).ti,ab. | keyword  TI/AB | 89.540 |
| 1 | 1A OR 1B |  | 250.074 |

## Block 2: intervention(s)

| **Search name** | **Search query** | **Type of search** | **Results** |
| --- | --- | --- | --- |
| 2A | ("potassium iodide" or "radioactive iodine").sh. | EMTREE headings  & subheadings | 13.298 |
| 2B | ("ITB" or "iodine thyroid blocking" or "potassium iodide" or "Iodine Radioisotope*" or "KI" or "sodium iodide" or ((blockade* or blocking or administration) and iodine) or "stable iodine" or ((prophylaxis or prophylactic* or "prophylactic agent*") and (iodine* or iodide*))).mp. | mp=title, abstract, heading word, drug trade name, original title, device manufacturer, drug manufacturer, device trade name, keyword | 71.537 |
| 2 | 2A OR 2B |  | 79.961 |

## Block 3: occurrence/location

| **Search name** | **Search query** | **Type of search** | **Results** |
| --- | --- | --- | --- |
| 3A | ("nuclear accident" or "radioactive waste" or "atomic warfare" or "Nuclear Reactor" or "Chernobyl accident" or "Nuclear Power Plant" or "Fukushima Nuclear Accident").sh. | EMTREE headings  & subheadings | 15.174 |
| 3B | (((Nuclear* or atomic or reactor* or radioactive* or radiation or radiological*) and (accident* or warfare or contaminat* or exposure* or fallout or meltdown or disaster* or catastrophe*)) or ((Belarus or chernobyl or Chornobyl or Hiroshima or Fukushima or Gomel or Homel or Ukraine or Minsk or "3 mile" or "three mile" or Nagasaki or Pripyat or Poland or Russia or USSR or "Soviet Union" or Japan) and (accident* or warfare or contaminat* or exposure* or fallout or meltdown or disaster* or catastrophe*))).mp. | mp=title, abstract, heading word, drug trade name, original title, device manufacturer, drug manufacturer, device trade name, keyword | 206.045 |
| 3 | 3A OR 3B |  | 210.269 |

## Limits

| **Search name** | **Search query** | **Results** |
| --- | --- | --- |
| 4 | elsevier.cr. | 15.768.140 |

## Summary & results

| **Search name** | **Results** |
| --- | --- |
| 1 AND 2 AND 3 | 1.339 |
| 1 AND 2 AND 3 AND 4 | 902 |

## Translation of subject headings from MeSH to EMTREE terms

## Block 1

| **MeSh Term** | **EMTREE Term** |
| --- | --- |
| thyroid gland | thyroid gland |
| hypothyroidism | hypothyroidism |
| thyroid diseases | thyroid disease |
| Thyroid neoplasms | **thyroid tumor** |
| neoplasms, radiation-induced | radiation induced neoplasm |
| radiation dosage | radiation dose |
| radiation injuries | radiation injury |
| dose-response relationship, radiation | **radiation response** |

## Block 2

| **MeSh Term** | **EMTREE Term** |
| --- | --- |
| Potassium Iodide | potassium iodide |
| Iodine Radioisotopes | **radioactive iodine** |

## Block 3

| **MeSh Term** | **EMTREE Term** |
| --- | --- |
| Radioactive Hazard Release | **Nuclear accident** |
| Radioactive Fallout | **Radioactive waste** |
| Nuclear Warfare | **atomic warfare** |
| Nuclear Reactors | Nuclear Reactor |
| Chernobyl Nuclear Accident | **Chernobyl accident** |
| Nuclear Power Plants | Nuclear Power Plant |
| Fukushima Nuclear Accident | Fukushima Nuclear Accident |
